# The Second Transmembrane Domain of P2X7 Contributes to Dilated Pore Formation

**DOI:** 10.1371/journal.pone.0061886

**Published:** 2013-04-17

**Authors:** Chengqun Sun, Michelle E. Heid, Peter A. Keyel, Russell D. Salter

**Affiliations:** Department of Immunology, University of Pittsburgh School of Medicine, Pittsburgh, Pennsylvania, United States of America; Institut Jacques Monod, France

## Abstract

Activation of the purinergic receptor P2X7 leads to the cellular permeability of low molecular weight cations. To determine which domains of P2X7 are necessary for this permeability, we exchanged either the C-terminus or portions of the second transmembrane domain (TM2) with those in P2X1 or P2X4. Replacement of the C-terminus of P2X7 with either P2X1 or P2X4 prevented surface expression of the chimeric receptor. Similarly, chimeric P2X7 containing TM2 from P2X1 or P2X4 had reduced surface expression and no permeability to cationic dyes. Exchanging the N-terminal 10 residues or C-terminal 14 residues of the P2X7 TM2 with the corresponding region of P2X1 TM2 partially restored surface expression and limited pore permeability. To further probe TM2 structure, we replaced single residues in P2X7 TM2 with those in P2X1 or P2X4. We identified multiple substitutions that drastically changed pore permeability without altering surface expression. Three substitutions (Q332P, Y336T, and Y343L) individually reduced pore formation as indicated by decreased dye uptake and also reduced membrane blebbing in response to ATP exposure. Three others substitutions, V335T, S342G, and S342A each enhanced dye uptake, membrane blebbing and cell death. Our results demonstrate a critical role for the TM2 domain of P2X7 in receptor function, and provide a structural basis for differences between purinergic receptors.

## Introduction

P2X7 is a receptor in the family of ATP-sensitive ionotropic purinergic P2X receptors, which consist of seven subtypes (P2X1-7). P2X receptors are typically homotrimeric, with each monomer containing two membrane spanning domains, an extracellular domain, and intracellular amino- and carboxy-termini [1]. P2X7 is expressed in many cell types, including cells from the hematopoietic lineages (erythrocytes, lymphocytes, neutrophils, eosinophils, mast cells, monocytes and macrophages), central and spinal cord neurons, brain glial cells (microglia, astrocytes and muller cells), bone cells (osteoblasts, osteoclasts and osteocytes), and epithelial and endothelial cells [2], [3], [4], [5], [6], [7], [8], [9], [10], [11], [12]. Expression of P2X7 has also been demonstrated in the enteric nervous system of the small intestine, kidney and urinary tract, uterus, and liver [13], [14], [15]. Activation of P2X7 mediates a number of physiological and pathological events including pore formation, phosphatidylserine exposure, membrane blebbing, phospholipase D and A_2_ activation, metalloproteinase activation, transmembrane protein shedding, pro-inflammatory cytokine maturation, caspase activation, apoptosis induction, pathogen killing, free radical production, cell cycle regulation, and T cell maturation [16], [17], [18], [19], [20], [21]. P2X7 is distinct from other P2X receptor subtypes in that P2X7 contains an extended 240 amino-acid C-terminal tail. The C-terminus is involved in mediating most downstream effects of P2X7, including pore-formation and signal transduction. For example, three loss of function single nucleotide polymorphisms (SNPs), T357S, E496A and I568N, and one gain of function SNP Q460R in human P2X7 are located in the C-terminus [22], [23], [24], [25]. These loss-of-function SNPs lead to reduced P2X7 pore formation and impaired ATP-induced mycobacterial killing by macrophages [23], [26], [27], [28]. Thus, the carboxyl terminal tail is thought to be responsible for the ability of P2X7 to form pores in the membrane following prolonged agonist stimulation [29].

Pore formation is one of the best studied characteristics of P2X7. Following brief activation by agonist, P2X7 forms a channel with strong selectivity for the divalent cations Ca^2+^ and Ba^2+^ over monovalent cations [30]. Continued stimulation by agonist results in the formation of a non-selective pore, which allows permeation of inorganic and organic cationic molecules up to 900 Da, such as N-methyl-D-glucamine, the monovalent cation ethidium bromide (Etd; cation mass 314Da), divalent cation propidium iodide (PI; cation mass 415 Da), and the divalent cation YoPro1 (cation mass 376 Da) [1], [31]. Due to this permeability, P2X receptor pore formation has been studied using these DNA-specific, cell impermeant fluorescent dyes [1], [29], [32], [33], [34], [35]. Although the divalent 279 Da cation DAPI is often used both in fixed and live cell staining because it is readily permeable to the small membrane pores induced by fixation [36], it has yet to be utilized to examine this larger P2X receptor non-selective pore. This pore formation is a trigger for inflammatory processes such as ATP-induced NLRP3 inflammasome activation and subsequent IL-1β cleavage and release by immune cells [37], [38]. It has been suggested that pore formation is not a unique feature of P2X7, but can also occur in cells expressing P2X2 and P2X4 [39], [40]. The pore itself could potentially be formed by an interacting protein, pannexin-1 [41], [42]. Since P2X7 is an ion channel, it is also possible that pore formation is an intrinsic property of P2X receptors, potentially involving conformational changes and/or recruitment of additional P2X subunits that result in dilation of the ion channel and corresponding increase in permeability [33], [43], [44]. Regardless of whether P2X7 itself forms the pore, it is clear that purinergic receptor activation is necessary for the initiation of the process. What structural determinants confer the ability of P2X7 to induce pore formation is unknown.

In this study, we tested the hypothesis that the C-terminal and TM2 domains of P2X7 confer dye permeability. We used the small cationic dye DAPI combined with the larger cationic dyes Etd, YoPro1 and PI to characterize the structural determinants of P2X7 pore formation. To address the role of P2X7 independently of pannexin-1, we transfected P2X7 wild type or mutant constructs into pannexin deficient NRK and C6 cells, as well as HEK293 cells. We found that exchanging either the P2X7 C-terminal domain or the second transmembrane domain (TM2) from P2X1 or P2X4 compromised surface expression and pore formation. Changing single amino acids to those present in P2X4 or P2X1 decreased dye uptake and membrane blebbing. Mutation of the predicted pore-lining residue S342 to that of P2X1 or P2X4 conferred sensitivity to pore formation, membrane blebbing and cell death. Taken all together, these data indicate the importance of TM2 for P2X7-mediated pore formation.

## Materials and Methods

### Cloning of cDNA and creation of mouse P2X receptor constructs

Total RNA was extracted and purified from mouse spleen cells (from C57BL/6 mice, provided by Lisa Borghesi, for P2X1 and P2X4 cloning) or FSDC cells [45] (provided by Paola Ricciardi Castagnoli, for P2X7 cloning) using RNeasy Mini Kit and RNase-Free DNase Set, as recommended by the manufacturer (Qiagen, Valencia, CA). MuLV Reverse Transcriptase (Life Technologies, Carlsbad, CA) was used to synthesize first strand cDNA from 1 µg of the purified total RNA at 42°C for 60 min using an oligo-dT_16–18_ primer (Life Technologies). A pair of sequence specific primers with the appropriate restriction enzymes sites were designed to amplify the entire coding sequence of the corresponding target cDNA genes by PCR, using AmpliTaq® DNA polymerase as described by the manufacturer (Life Technologies). PCR products were separated on a 1% agarose gel containing 1 µg/ml ethidium bromide (Sigma, Saint Louis, MO), purified with Wizard® SV Gel and PCR Clean-Up System (Promega, Madison, WI) and cloned into the pFB-Neo Retroviral vectors (Stratagene, La Jolla, CA), using the appropriate restriction enzymes and the Rapid DNA ligation Kit (Roche Applied Science, Indianapolis, IN, USA). pFB-Neo has neomycin-resistant sequences in which the multiple cloning sites were modified. Since FSDC are of C57BL/6 origin, a strain with low P2X7 function, we introduced a substitution in our P2X7 construct by Quikchange mutagenesis (Agilent Technologies, Santa Clara, CA) corresponding to the Balb/C allele, which has normal function. The translated amino acid sequences of the P2X1, P2X4, and P2X7 constructs we used for transfection and mutagenesis are presented in Figure S1. Detailed plasmid maps and sequences of primers are available upon request. All of the vectors and mutants were verified by DNA sequencing.

An overlapping PCR strategy and/or fragment swapping with appropriate restriction enzymes was used for mutagenesis of the P2X7 constructs. Amplicons containing the mutations were subcloned into the corresponding regions of the P2X7 constructs using the appropriate restriction enzymes and Rapid DNA ligation Kit. The expand high fidelity PCR system (Roche Applied Science, Indianapolis, IN) was used for all the overlapping PCR reactions. Detailed plasmid maps and sequences of primers are available upon request. All of the vectors and mutants were verified by DNA sequencing.

### Cell culture

Human embryonic kidney (HEK) 293 cells (ATCC, Manassas, VA catalog number CRL-1573), normal rat kidney (NRK) cells (ATCC, CRL-6509), and C6 glioma cells (ATCC, CCL-107) were grown at 37°C, 5% CO_2_ in D10 (Dulbecco's modified Eagle medium (DMEM) supplemented with 10% fetal bovine serum (FBS), 100 units/ml penicillin and 100 µg/ml streptomycin). Retroviral packaging gp293 cells (Clontech, Palo Alto, CA) expressing gag/pol proteins were also cultured in D10.

### Transfection and production of VSV-pseudotyped retroviruses

gp293 cells were plated in six-well plates one day before transfection and co-transfected with a P2X construct and VSV-G using Lipofectamine LTX Reagent, as recommended by the manufacturer (Life Technologies) using the following amounts and volumes: 4 µg of P2X construct DNA and 2 µg of VSV-G DNA, 5 µl of Plus reagent, and 10 µl of Lipofectamine LTX Reagent in 500 µl of Opti-MEM I were used per well. Two days after transfection, the media containing the retrovirus was collected, filtered and stored at −80°C until use.

### Transduction and selection of stable expression cell lines

2×10^4^ cells/well of HEK293, NRK, or C6 cells were seeded in 12-well one day before retrovirus infection. Cells were infected with the recombinant VSV-pseudotyped retrovirus. At 24 hours post infection, 1 mg/ml G418 was added to the culture media. After two weeks of selection, G418-resistant cells were expanded and maintained in 0.5 mg/ml G418 for assays.

### Flow cytometry

To monitor the surface expression of the P2X7 and mutants, cells were stained with rat anti- mouse P2X7 monoclonal antibody (mAb) HANO43 (ALEXIS, San Diego, CA) using standard FACS procedures. In brief, transduced cells were harvested with Trypsin-EDTA (Life Technologies), collected by centrifugation, washed three times with FACS wash buffer (5% FBS and 0.5 mg/L sodium azide in PBS), and re-suspended in PBA buffer (1% BSA and 0.2 mg/L sodium azide in PBS) containing 0.5% normal human serum for 10 min at 4°C. The cells were then washed one time with PBA. The cells were re-suspended with PBA with the anti-P2X7 mAb and incubated on ice for 40 min. Pilot experiments were performed with titrations to determine the amount of HANO43 required for maximal binding. The cells were washed three times with FACS swash buffer, re-suspended in PBA with cy5-conjugated anti-rat Ig-G antibody (Jackson ImmunoResearch Laboratories, West Grove, PA) and incubated on ice while protected from light for 30 min. The cells were then washed three times with the FACS wash buffer and fixed with 2% paraformaldehyde in PBS before analysis with BD LSRII flow cytometer (BD Biosciences, San Jose, CA). A total of 2×10^4^ cells/sample were acquired and analyzed by using FlowJo software (Treestar, Ashland, OR).

### Dye uptake

Flow cytometry was used to determine the uptake of DAPI, Etd, YoPro1 (4-[(3-methyl-2-(3H)-benzoxazolylidene) methyl]-1-[3-(triethylammonio) propyl]diiodide), and PI in retrovirally-transduced cells. Briefly, cells were dislodged using Trypsin-EDTA and washed with D10. Some of the cells were surface stained for each P2X7 construct. About 5×10^5^ cells in D10 with 3.6 µM DAPI, 5 µM PI, 20 µM Etd and/or 2.5 µM YoPro1 and with various concentrations of ATP (Sigma, Saint Louis, MO) were incubated at 37°C, 5% CO_2_ for 30 min. Then cells treated with ATP were washed with cold PBS and re-suspended with PBS prior to analysis on the LSRII. A total of 1×10^4^ cells/sample were acquired and analyzed using FlowJo. The P2X7 inhibitors A74003 and A438079 were from Tocris BioScience (Ellisville, MO) and were used at concentrations of 100 µM and 10 µM respectively.

### Western blotting

Cell lysates of stably transduced lines were prepared with NP-40 lysis buffer (Boston BioProducts, Worcester, MA) supplemented with a protease inhibitor cocktail (Calbiochem, La Jolla, CA). For each lysate, 30 µg was resolved on an 11% SDS-PAGE, transferred to PVDF and stained with appropriate primary and HRP-conjugated secondary antibodies. The membrane was imaged on an Image Station 4000 MM (Kodak, Rochester, NY) using Western Blotting Luminol Reagents (Santa Cruz Biotechnology, Santa Cruz, CA).

### Live cell imaging

Transduced NRK cells were plated at 2.5×10^5^ cells per dish one day prior to imaging in glass bottom dishes (Mattek Corporation, Ashland, MA). Cells were imaged every 30 seconds in the presence of 3.6 µM DAPI, 5 µM PI and 3 mM ATP for 45–60 min on a Nikon A1 confocal microscope equipped with appropriate excitation and emission filters using Elements (Nikon, Melville, NY). Images were exported as tif files and processed in Metamorph (Molecular Devices, Sunnyvale, CA) using Equalize Light and Median Pass Filter functions.

## Results

### P2X7 mediates rapid DAPI uptake

**Figure 1:**
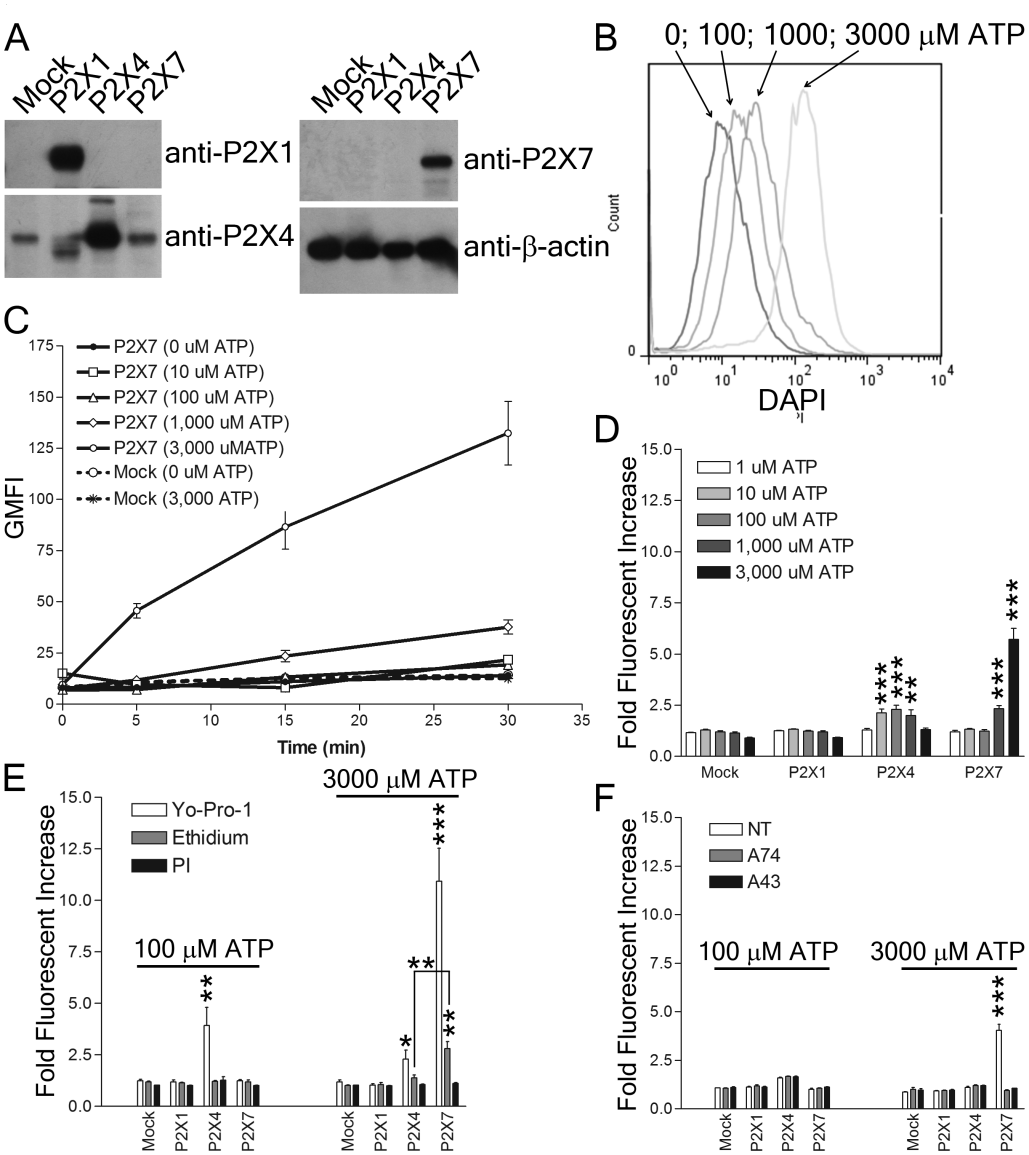
P2X7 mediates rapid ATP-dependent DAPI uptake. (A) NRK cell lysates (30 µg/lane) stably expressing P2X1, P2X4, P2X7 or vector alone (mock) were resolved by SDS-PAGE, transferred to PVDF and membranes probed with the indicated antibodies. (B) NRK cells stably expressing P2X7 were treated with the indicated concentrations ATP for 15 min and DAPI uptake analyzed by flow cytometry. (C) NRK cells stably expressing either P2X7 or vector alone (mock) were incubated with the indicated concentrations of ATP for the indicated times, and DAPI uptake measured by flow cytometry. The geometric mean fluorescence intensity (GMFI) is shown. (D, E) NRK cells stably expressing P2X1, P2X4, P2X7 or vector alone (mock) were treated with ATP for 15 min and uptake of DAPI (D), Etd, YoPro1, and PI (E) measured by flow cytometry. Fold fluorescent increase was determined by dividing the GMFI of cells in the presence of ATP by the GMFI in the same cells without addition of ATP (GMFI_ATP_/GMFI_no-ATP_). (F) NRK cells stably expressing P2X1, P2X4, P2X7 or vector alone (mock) were treated with ATP for 15 min in the absence (NT) or presence of 100 µM A74003 (A74) or 10 µM A438079 (A43) and uptake of DAPI was measured by flow cytometry. The data shown are representative of more than three independent experiments and two tailed Student's t-tests (unpaired) compared to mock yielded p values <0.05 (*), <0.01 (**), or <0.001(***).

We used DAPI to evaluate the ability of P2X receptors to form pores in response to ATP because it is a smaller divalent cation than previously used dyes and likely to be more sensitive to pore formation. To test DAPI uptake mediated by P2X receptors, we expressed them in NRK cells by retroviral transduction. High levels of P2X1, P2X4, and P2X7 were detected after transduction by western blot, which also revealed low endogenous levels of P2X4 in NRK cells (Fig. 1A). Following treatment with titrated amounts of ATP, we found that P2X7 mediated rapid DAPI uptake in a dose- and time-dependent fashion (Fig. 1B, C). There was a clear distinction in DAPI uptake induced by ATP by the different P2X receptors (*p* = 0.0001 by analysis of variance for data derived from 15 min incubation with ATP) (Fig. 1D). Consistent with the P2X receptors showing different sensitivities to ATP, cells expressing P2X7 showed DAPI uptake at high concentrations of ATP (1 mM and 3 mM) while those overexpressing P2X4 showed DAPI uptake at moderate concentrations of ATP (0.01–1 mM) (Fig. 1D). P2X1 did not induce any observable DAPI uptake (Fig. 1D). YoPro1 uptake was similar to DAPI uptake in that cells expressing P2X4 accumulated YoPro1 following stimulation with 100 µM ATP while cells expressing P2X7 took up YoPro1 following stimulation with 3 mM ATP (Fig. 1E). Further experiments suggested that increasing dye size and/or reducing dye charge reduced P2X receptor pore function. P2X4 did not mediate the uptake of either Etd or PI, while P2X7expressing cells accumulated low levels of Etd but no significant amount of PI (Fig. 1E). Treatment of the cells with P2X7 inhibitors A740003 or A438079 blocked DAPI uptake, indicating that DAPI uptake required P2X7 function (Fig. 1F). These data indicate that DAPI uptake serves as a sensitive measure of P2X receptor dependent pore formation.

### P2X7 C-terminus is required for efficient surface expression and dye uptake

**Figure 2:**
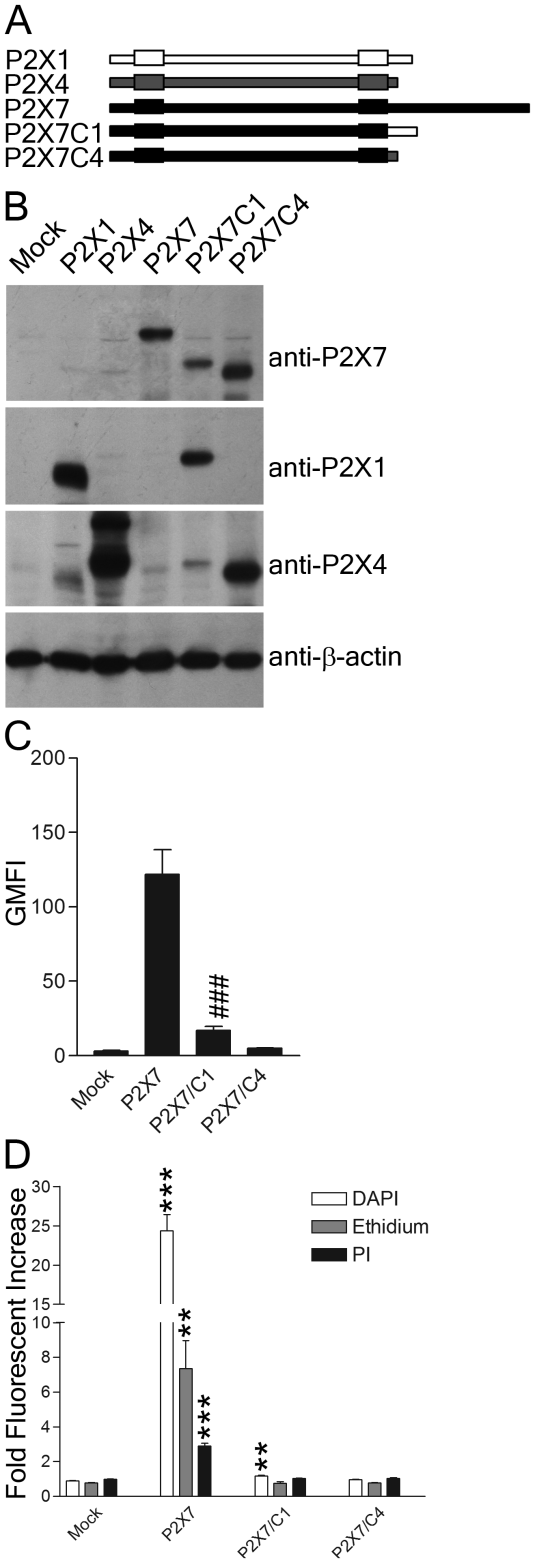
The C-termini of P2X1 and P2X4 do not substitute for the C-terminus of P2X7. (A) Schematic representation of chimeric constructs generated by swapping the C-termini of P2X receptors. P2X7C1 is P2X7 where the C-terminus swapped for P2X1, while P2X7C4 is P2X7 where the C-terminus has been swapped for P2X4. (B) HEK293 cell lysates (30 µg/lane) stably expressing vector alone (mock), P2X1, P2X4, P2X7, P2X7C1 or P2X7C4 were resolved by SDS-PAGE, transferred to PVDF and membranes probed with the indicated antibodies. (C, D) HEK293 cells stably expressing vector alone (mock), P2X7, P2X7C1 or P2X7C4 were either (C) surface stained with the anti-P2X7 mAb HANO43 or (D) treated with 3 mM ATP for 30 min in the presence of DAPI, Etd and PI and analyzed by flow cytometry. The data shown are representative of more than three independent experiments and two tailed Student's t-tests (unpaired) compared to wild type P2X7 yielded p values <0.001 (###), while comparisons to mock yielded p values <0.01 (**), or <0.001 (***).

The 240aa C-terminal domain of P2X7 is longer than that of other P2X receptors and is important for regulating pore formation. [29], [46]. To identify discrete functional regions within this larger domain, we generated a series of truncated P2X7 constructs and transduced them into HEK293 and NRK cells. While individual mutant proteins were detected in cells via western blot, they did not reach the surface and did not mediate any dye uptake (unpublished data). To further characterize the role of the C-terminus in P2X7 pore formation, we constructed two chimeric P2X7 receptors by exchanging the C-terminus of P2X7 with that of either P2X1 (P2X7C1) or P2X4 (P2X7C4) (Fig. 2A). Both P2X7C1 and P2X7C4 were detected as proteins in HEK293 cells (Fig. 2B). Surface expression was tested using the anti-P2X7 mAb HANO43. The C-terminus of P2X1 was sufficient to partially rescue surface expression of the chimeric P2X7C1, though the C-terminus of P2X4 was not (Fig. 2C). Cells expressing the chimeric constructs were compared to wild type P2X7 and mock transduced cells for their ability to internalize to DAPI, Etd, and PI following stimulation by 3 mM ATP (Fig. 2D). Dye uptake was absent in these transduced cells. P2X7C1 did internalize low levels of DAPI relative to mock, though these levels were lower than one might predict based on surface expression (Fig. 2D). In contrast to NRK cells, P2X7 induced PI uptake in the transduced HEK293 cells (Fig. 1E vs Fig. 2D). These data illustrate that the C-terminus of P2X7 is crucial for proper surface expression of P2X7.

### The second transmembrane domain (TM2) of P2X7 is required for pore formation

**Figure 3:**
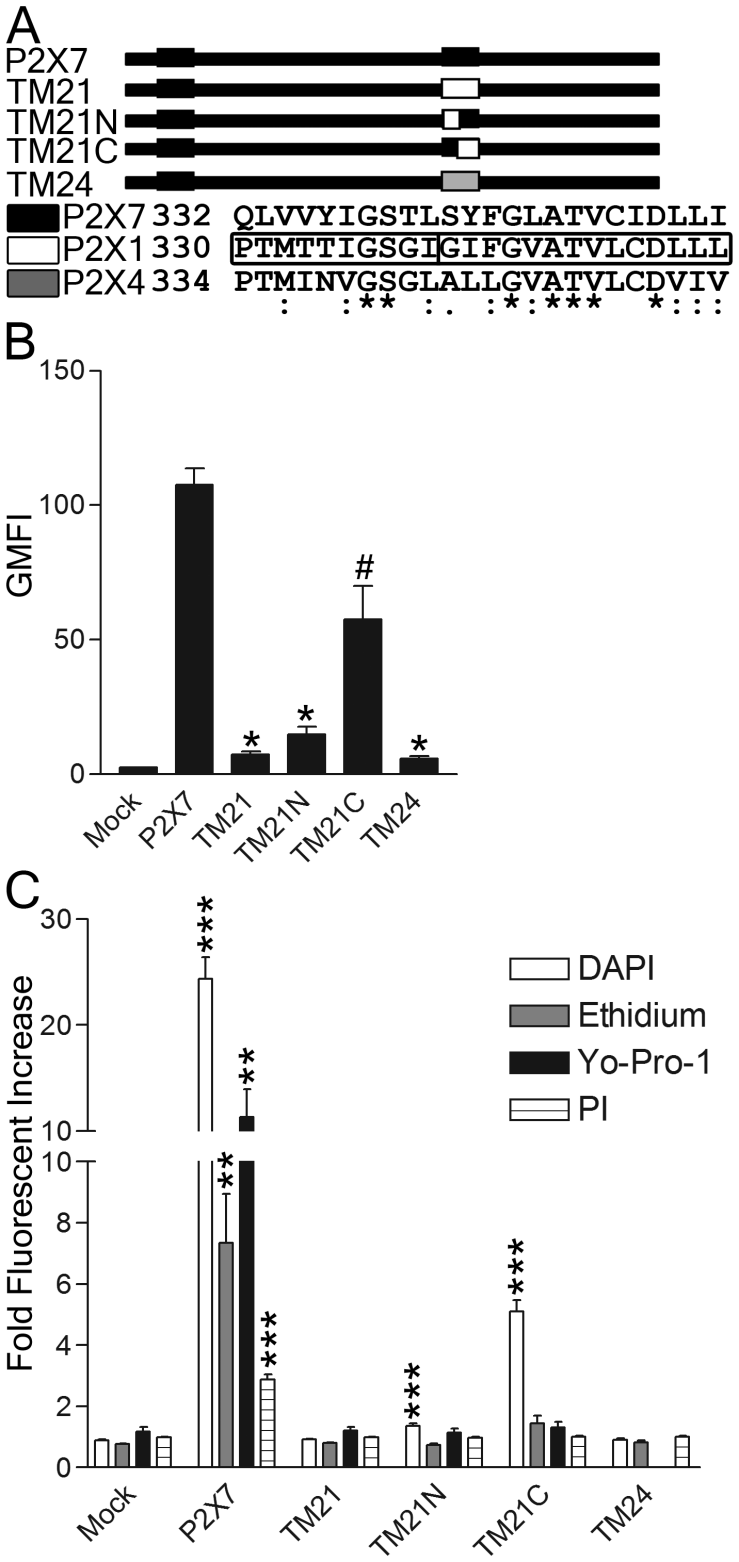
The TM2 of P2X7 confers surface expression and channel activity. (A) Schematic representation of the chimeric constructs used. All constructs are P2X7 with either the entire TM2 replaced with that of P2X1 (TM21), that of P2X4 (TM24) or the first or second half of TM2 replaced with that of P2X1 (TM21N and TM21C, respectively). A sequence alignment of P2X1, P2X4 and P2X7 are shown with *, :and. indicating identical, conserved and similar residues, respectively. (B, C) HEK293 cells stably expressing the vector alone (mock), P2X7 or the chimeric constructs were either (B) surface stained with anti-P2X7 mAb HANO43 or (C) treated with 3 mM ATP for 30 min in the presence of DAPI, Etd, YoPro1 and PI and analyzed by flow cytometry. The data shown are representative of more than three independent experiments and two tailed Student's t-tests (unpaired) compared to mock yielded p values of <0.05 (*), <0.01 (**), or <0.001(***) while comparisons to wild type P2X7 yielded p values <0.05 (#).

We next examined whether the second transmembrane domain (TM2) of P2X7 is required for pore formation. In zebrafish P2X4.1 receptor (zfP2X4), three TM2 α-helices combine to form the ion conducting pathway, using one TM2 from each subunit of the trimer [47]. The pore of other P2X receptors is also lined by residues within TM2, with TM1 making little contribution to ion flow [48], [49], [50], [51], [52], [53]. Based on alignment of amino acid sequences in TM2 from available P2X receptors, P2X7 has higher sequence identity to P2X1 (45.8%) and P2X4 (33.3%) than to zfP2X4 (29.2%) and P2X2 (12.5%). To determine the importance of P2X7 TM2 in pore formation, we generated chimeric P2X7 receptors where TM2 was replaced by that of P2X1 and P2X4 (Fig. 3A). Expression of the chimeric constructs was confirmed by western blotting (unpublished data). All of the chimeric constructs were expressed on the surface, though the chimeras had reduced surface expression (Fig. 3B). When the TM2 domain of P2X7 was swapped with either P2X1 or P2X4, dye uptake was abolished, potentially due to low surface expression (Fig. 3C). We generated two more chimeric constructs where the C-terminal or N-terminal portion of P2X1 replaced that of P2X7. When the C-terminus of P2X1 was substituted into P2X7, surface expression was much higher than in other chimeric constructs (Fig. 3B). Interestingly, DAPI uptake persisted, though at a reduced level, while uptake of the larger dyes was abolished (Fig. 3C). Thus, TM2 is critical for surface expression and pore formation of P2X7.

### Single amino acid substitutions in TM2 control surface expression and pore formation

To further compare the TM2 of P2X7 with that of P2X1 and P2X4, we introduced single amino acid substitutions between positions 332–343. One of these, the mutation S342F confers ATP resistance to RAW cells [54]. This mutation is predicted to almost completely obstruct the pore, as shown recently by Browne et al [55] and may interfere with successful folding or trimerization. S342 is also the most divergent residue predicted to line the pore amongst the P2X receptors, as the other residues are either identical or very similar, except C350, which is predicted to lie furthest away from the narrowest portion of the pore. Although we observed decreased dye uptake when substituting P2X1 or P2X4 TM2 for P2X7, substituting S342 for the corresponding residue in either P2X1 or P2X4, might be expected to result in a pore that is slightly larger and less charged.

**Figure 4:**
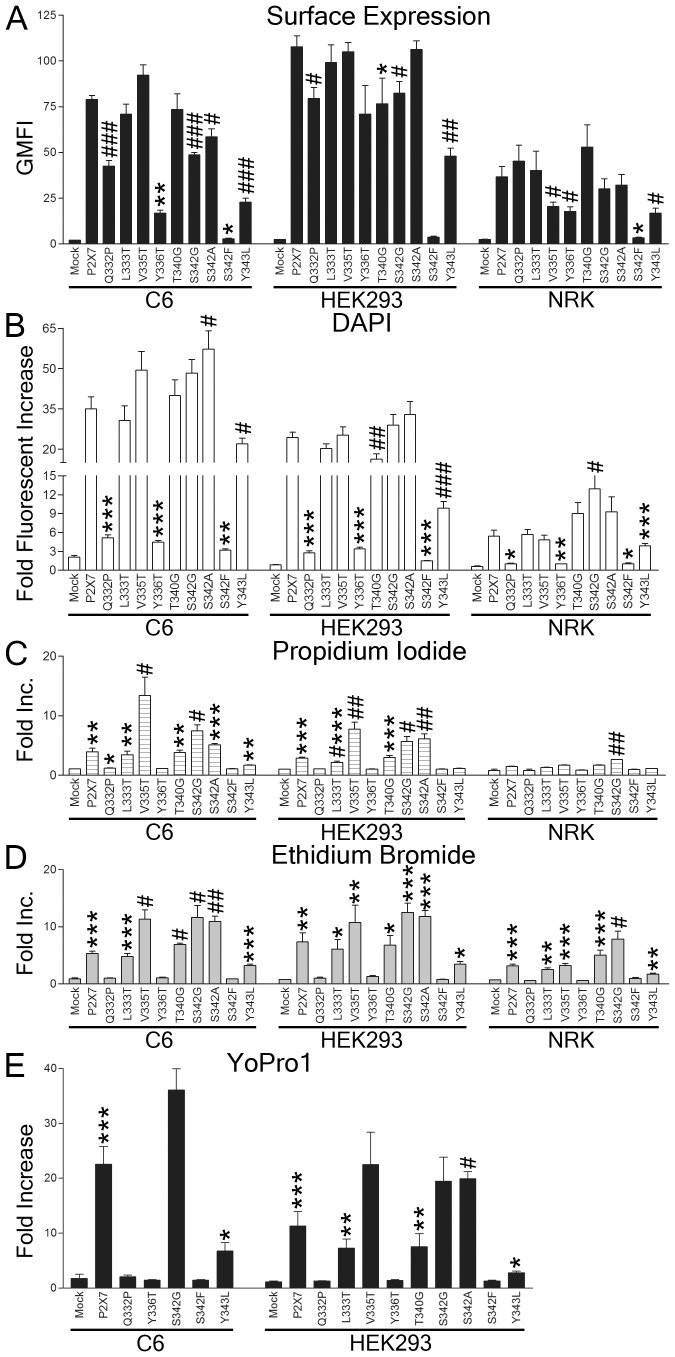
Point mutations of P2X7 TM2 domain alter dye permeability. The indicated cell lines were transduced with either vector alone (mock), wild type P2X7 or P2X7 expressing the indicated mutations and either (A) surface stained with anti-P2X7 mAb HANO43, or treated with 3 mM ATP for 30 min in the presence of (B) DAPI, (C) PI, (D) Etd, or (E) YoPro1 and analyzed by flow cytometry. GMFI was determined for live transduced cells. The data shown are representative of more than three independent experiments and two tailed Student's t-tests (unpaired) compared to wild type P2X7 yielded p values <0.05 (#), <0.01 (##), or <0.001(##), and compared to mock yielded p values of <0.05 (*), <0.01 (**) or <0.001(***).

Since the pore-lining residues are highly conserved between P2X receptors, we hypothesized that the residues not lining the pore, which are more variable between P2X receptors, are more likely to govern pore permeability. Based on our alignment of the P2X receptors (Fig. 3A), we selected eight single residues within TM2 to mutate to the corresponding residue in either P2X1 or P2X4. Three of the substitutions (Q332P, L333T, T340G) matched residues conserved between P2X1 and P2X4, while two matched the P2X1-specific residues (V335T, Y336T) and one matched a P2X4-specific residue (Y343L). We also mutated the pore-lining S342 to the corresponding residue in either P2X1 or P2X4, as well as to the ATP-resistant S342F mutation. We transduced HEK293, NRK, and C6 cells with these P2X7 constructs and measured surface expression and dye uptake. All mutants were expressed on the cell surface, except S342F, which only expressed at extremely low levels on C6 and NRK cells (Fig. 4A). We found that Y336T and Y343L had reduced surface expression in all three cell types compared to the wild type P2X7 (Fig. 4A). All of the constructs mediated some degree of DAPI uptake in the transduced C6, HEK293, NRK cells (Fig. 4B). Compared to wild type P2X7, Q332P, Y336T, and S342F had greatly reduced DAPI uptake, while S342A in C6 cells and S342G in NRK cells showed increased DAPI uptake (Fig. 4B). Similarly, no PI uptake was observed in the cells transduced with Q332P, Y336T, S342F, and Y343L while S342G promoted increased PI uptake (Figure 4C). Significant PI uptake was also found in the C6 and HEK293 cells transduced with L333T, V335T, T340G, S342A, and wild type P2X7 (Fig. 4D). Similar results to PI were obtained using Etd and YoPro1, except Y343L (Fig. 4E–F). Cells with Y343L mutant showed uptake of both Etd and YoPro1 (Fig. 4E–F). Taken together, these dye uptake assays indicate that positions 332, 336, 342, and 343 in the TM2 region are responsible for regulating pore formation in P2X7. It should be noted that the Y336T mutation is expressed at reduced levels particularly in C6 transfectants, and that this might account at least in part for impaired dye uptake by these cells.

### P2X7-mediated blebbing and death requires residues important for pore permeability

**Figure 5:**
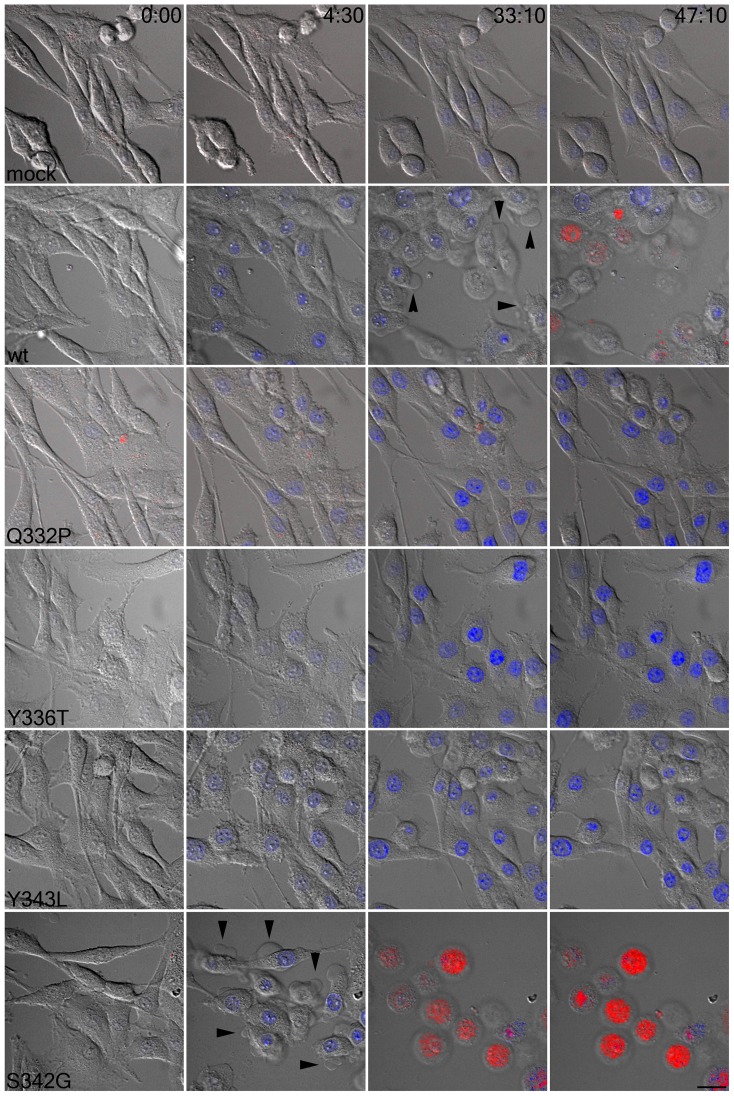
Point mutations of P2X7 TM2 alter cell viability. NRK cells stably transduced with the indicated constructs were imaged at 2 frames/minute for 45 minutes in the presence of DAPI (blue) and PI (red) following the addition of 3 mM ATP. Arrowheads indicate blebs. Images shown are representative of at least 10 fields of cells from at least two independent experiments. Scale bar = 20 µm.

To determine whether TM2 substitutions influenced downstream effects of P2X7, such as plasma membrane blebbing, we exposed transduced NRK cells to 3 mM ATP in the presence of DAPI and PI. Live cell imaging was used to detect changes in cell morphology and in dye uptake. Mock-transduced cells showed slow P2X7-independent DAPI uptake and no membrane blebbing or PI uptake following addition of 3 mM ATP (Fig. 5). Wild type P2X7 exhibited rapid DAPI uptake and robust membrane blebbing, finally resulting in PI uptake and cell death (Fig. 5). In contrast Q332P, Y336T, and Y343L mediated DAPI uptake and minor membrane blebbing, but no PI uptake or cell death (Fig. 5). The S342G which showed increased DAPI and PI uptake also showed more robust membrane blebbing and PI uptake than wild type P2X7 (Fig. 5). Whereas blebbing in wild type P2X7 transduced cells was evident at 33 min and death by 45 min, cells bearing the S342G mutation showed blebbing as early as 5 min and death by 30 min (Fig. 5). Thus, the kinetics of P2X7-mediated membrane blebbing correlates with the ATP-gated pore formation of P2X7.

## Discussion

Here we compared the ability of three P2X receptors, P2X1, P2X4 and P2X7 to promote ATP-dependent pore formation. P2X1 did not promote pore formation, while P2X4 did so only at low ATP concentrations. To determine what unique structural requirements permit P2X7 to induce pores, we generated chimeric P2X receptors. We found that the C-terminus of P2X7 was necessary for surface expression while TM2 was necessary for surface expression and pore formation. Specifically within TM2, we identify four amino acid residues unique to P2X7 that are needed for pore formation, and in which substitutions are deleterious to function, as demonstrated by altered membrane blebbing and cell death. An additional position, S342, is also critical to function, and substitution of a less bulky side group (glycine) results in a hyperactive receptor with enhanced capacity for mediating dye uptake, membrane blebbing, and cell death.

The ability of P2X7 receptor to form a pore has been questioned previously since an associated protein, pannexin-1, has been shown to mediate dye uptake following ATP stimulation [41], [42]. However, there is strong evidence that P2X2, P2X4, and P2X7, can autonomously form dilated pore structures [1], [39], [40]. To exclude a role for pannexin-1 in our system, we used two cells lines for transfection, NRK and C6, which lack pannexin-1 [56], [57]. A third line, HEK293, expresses pannexin-1 based on RT-PCR analysis, but we have no evidence that it is functional since panx10, a peptide mimetic, does not block dye uptake in P2X7 transfectants (data not shown).

Previously, it has been reported that only homomeric P2X2, P2X4, and P2X7 can form integral pores during the prolonged agonist application, but not the other P2X receptors [1], [39], [40]. Consistent with these data, we find that P2X4 and P2X7 can mediate rapid DAPI uptake and YoPro1 uptake, but P2X1 could not. Since truncated P2X7 receptors lack dye uptake but retain ion channel activities [46], we generated a series of P2X7 receptors where the C-terminus was lacking. These constructs did not reach the cell surface, and were presumed to be non-functional. We next tested whether the C-terminus from either P2X1 or P2X4 could rescue surface expression. Neither of these chimeras rescued surface expression, indicating that the long P2X7 C-terminus is required for its efficient surface expression.

To further probe the specific ability of P2X7 to form pores, we examined the TM2 region. Most structural information on TM2 has been derived from studies on P2X2 and P2X4. The crystal structure of the closed zP2X4 receptor directly reveals that the ion-conducting pathway is formed by three TM2 α-helices, each provided by one subunit of the trimer [47]. The P2X pore is lined by TM2, with TM1 making little contribution to ion flow [51], [58]. TM2 is also involved in protein folding and assembly of P2X subunits [59], [60]. When we exchanged the TM2 of P2X7 for that of either P2X1 or P2X4, we found highly reduced surface expression and no pore formation. This implies that the intrinsic fine amino acid structure of TM2 might be necessary for the folding and function of P2X receptors. Given that P2X4 itself can form a large, permeable pore, the domains may require unique residues on other domains within the same protein [39], [61]. To avoid this potential problem, we swapped smaller portions of TM2, either the N-terminal or C-terminal domains of the P2X7 TM2 with that of the pore-incompetent P2X1 (TM21N and TM21C constructs). TM21C restored surface expression and displayed limited pore permeability to DAPI. The homology between the P2X1 and P2X7 C-terminal fourteen residues of TM2 (57%) is much higher than that of the N-terminal ten residues (30%). This may account for the higher surface expression and DAPI uptake we obtained in cells transduced with TM21C over those transduced with TM21N. Neither chimeric construct permitted uptake of larger dyes. This may be due to the location of the gate region within P2X receptors. Previous studies on P2X2 and P2X4 have suggested that the gate region is located in the outer half of TM2 in the closed state [47], [51], [61], [62].

To further map the structural determinants of pore formation, we examined individual residues within TM2. The TM2 helices cross in the outer leaflet of the membrane, giving the pore an hourglass appearance, with the narrowest constriction formed by three residues in the TM2 helix: L340, A344 and A347 in zfP2X4 and I332, T336, and T339 in rat P2X4 [47], [53], [62]. The residues at the corresponding positions in P2X1 and P2X7 are T333, S337 and G340 in murine P2X1 and V335, S339 and S342 in murine P2X7. Recently, homology models of rP2X2 and hP2X7 have been generated based on the crystal structure of the zfP2X4 [53], [63]. These models predict that residues I332, T336, T339, V343, and D349 line the pore in the open conformation of rP2X2 [53]. The point mutations we made in V335 and S342 in mouse P2X7 correspond with I332 and T339 of rP2X2. We found that mutation of either residue to the corresponding one in P2X1 enhanced pore formation, consistent with these residues residing near the pore. Mutation of S342F, which confers ATP-resistance in RAW cells [54], resulted in nearly complete loss of surface expression and pore formation of P2X7. This may be due to poor trimerization or folding of this mutant P2X7. Taken together, our data suggest certain residues may be important for pore formation in P2X7.

We found that V335T, S342G, and S342A enhanced dye uptake, without significant increase in surface expression. Although S342 is thought to be the narrowest part of the pore [55], V335T may not be required for initial P2X7 pore formation, but rather for the transition from the small ion and DAPI permeable pore to the larger pore. Similarly, the residue A348 in hP2X7 is predicted to be part of intracellular pore and close to the physical gate that occludes the ion permeating pathway in the closed state [53]. A348T is a gain of function SNP (A348T) that affects the pore formation function and enhances IL-1β release [34], [63], [64], which we predict would be similar to that observed for V335T. In contrast to V335, we predict that S342 does line the pore of P2X7. We find that mutation to either the non-polar alanine or the much smaller glycine enhances P2X7 pore activity to the extent that cells with a mutant receptor bleb and die much faster than cells expressing wild type P2X7. We conclude that residues near the pore may alter pore sensitivity.

Substitution of three other residues for those in P2X1 or P2X4 (Q332P, Y336T, and Y343L) limited the ATP-gated pore formation in the transduced cells. These mutants exhibited a decrease in DAPI uptake, larger dye uptake and membrane blebbing, indicating they had compromised pore-activity. While it is possible these residues could indirectly contribute the size of the pore, we cannot rule out that they are needed for interacting with other proteins or portions of P2X7. Interestingly, substitution of G347Y in P2X4 (corresponds to G345 in P2X7) abolishes permeability to NMDG [39]. Thus, we have identified residues important for full P2X7 function.

## Supporting Information

Figure S1 **Translated amino acid sequences of the P2X constructs used in this study.** Details of cloning procedures for isolating cDNAs and vector use are described in the Methods section.
(DOC)
