# Supplementary material for: The Second Transmembrane Domain of P2X7 Contributes to Dilated Pore Formation
Source: PLoS One. 2013 Apr 17;8(4):e61886. doi: 10.1371/journal.pone.0061886 (PMC3629090; doi:10.1371/journal.pone.0061886)
Supplement: Figure S1 — Translated amino acid sequences of the P2X constructs used in this study. Details of cloning procedures for isolating cDNAs and vector use are described in the Methods section. (DOC) [file pone.0061886.s001.doc]

P2X1

MARRLQDELSAFFFEYDTPRMVLVRNKKVGVIFRLIQLVVLVYVIGWVFVYEKGYQTSSGLISSVSVKLKGLAVTQLQGLGPQVWDVADYVFPAHGDSSFVVMTNFIMTPQQAQGHCAENPEGGICQDDSGCTPGKAERKAQGIRTGNCVPFNGTVKTCEIFGWCPVEVDDKIPSPALLHEAENFTLFIKNSISFPRFKVNRRNLVEEVNGTYMKKCLYHKILHPLCPVFSLGYVVRESGQDFRSLAEKGGVVGITIDWECDLDWHVRHCKPIYQFHGLYGEKNLSPGFNFRFARHFVQNGTNRRHLFKVFGIRFDILVDGKAGKFDIIPTMTTIGSGIGIFGVATVLCDLLLLHILPKRHYYKQKKFKYAEDMGPGEGERDPAATSSTLGLQENMRTS

P2X4

MAGCCSVLRAFLFEYDTPRIVLIRSRKVGLMNRVVQLLILAYVIGWVFVWEKGYQETDSVVSSVTTKAKGVAVTNTSQLGFRIWDVADYVVPAQEENSLFIMTNMIVTVNQTQGTCPEIPDKTSICDSDANCTLGSSDTHSSGIGTGRCVPFNASVKTCEVAAWCPVENDAGVPTPAFLKAAENFTLLVKNNIWYPKFNFSKRNILPNITTSYLKSCIYNARTDPFCPIFRLGQIVADAGHSFQEMAVEGGIMGIQIKWDCNLDRAASHCLPRYSFRRLDTRDLEHNVSPGYNFRFAKYYRDLAGNEQRTLTKAYGIRFDIIVFGKAGKFDIIPTMINVGSGLALLGVATVLCDVIVLYCMKKRYYYRDKKYKYVEDYEQGLSGETDQ

P2X7

MDYKDDDDKDYKDDDDKPACCSWNDVLQYETNKVTRIQSTNYGTVKWVLHMIVFSYISFALVSDKLYQRKEPVISSVHTKVKGIAEVTENVTEGGVTKLGHSIFDTADYTFPLQGNSFFVMTNYVKSEGQVQTLCPEYPRRGAQCSSDRRCKKGWMDPQSKGIQTGRCVPYDKTRKTCEVSAWCPTEEEKEAPRPALLRSAENFTVLIKNNIHFPGHNYTTRNILPTMNGSCTFHKTWDPQCSIFRLGDIFQEAGENFTEVAVQGGIMGIEIYWDCNLDSWSHHCRPRYSFRRLDDKNTDESFVPGYNFRYAKYYKENNVEKRTLIKAFGIRFDILVFGTGGKFDIIQLVVYIGSTLSYFGLATVCIDLLINTYSSAFCRSGVYPYCKCCEPCTVNEYYYRKKCESIMEPKPTLKYVSFVDEPHIRMVDQQLLGKSLQVVKGQEVPRPQMDFSDLSRLSLSLHDSPPTPGQSEEIQLLHEEVAPKSGDSPSWCQCGNCLPSRLPEQRRALEELCCRRKPGRCITTSKLFHKLVLSRDTLQLLLLYQDPLLVLGEEATNSRLRHRAYRCYATWRFGSQDMADFAILPSCCRWRIRKEFPKTEGQYSGFKYPY
